# Commodification and healthcare in the third sector in England: from gift to commodity—and back?

**DOI:** 10.1080/09540962.2023.2244350

**Published:** 2023-08-15

**Authors:** Rod Sheaff, Angela Ellis-Paine, Mark Exworthy, Rebecca Hardwick, Chris Q. Smith

**Affiliations:** a Peninsular School of Medicine and Dentistry, University of Plymouth, UK; b Bayes Business School, City University London; c Health Services Management Centre, University of Birmingham, UK

**Keywords:** Commissioning, commodification, England, inter-organization collaboration, networks, outsourcing, third sector, Washington consensus

## Abstract

**IMPACT:**

This article suggests why a different approach may be required for commissioning services from third sector providers than from, say, corporate or public providers. English systems for commissioning third sector providers contain both commodified elements (for example formal procurement, provider competition, commissioner–provider separation) and collaborative, relational elements (for example long-term collaboration, reliance on inter-organizational networks). When the two elements conflicted, commissioners and third sector organizations tended to try to work around the commodified elements in order to preserve and develop the collaborative aspects, which suggests that, in practice, they find de-commodified, collaborative methods better adapted to the commissioning of third sector organizations.

**ABSTRACT:**

When publicly-funded services are outsourced, governments still use multiple governance structures to retain some control over the services provided. Using realist methods the authors systematically compared this aspect of community health activities provided by third sector organizations in six English localities during 2020–2022. Two modes of commissioning coexisted. Commodified commissioning largely embodied Washington consensus models of formal, competitive procurement. A contrasting, collaborative mode of commissioning relied more upon relational, long-term co-operation and networking among organizations. When the two modes conflicted, commissioners often favoured the collaborative mode and sought to adjust their commissioning to make it less commodified.

When publicly-funded services become commodified and provided by corporations or third sector organizations, governments nevertheless wish to retain some control over the services provided. To do so, governments often use multiple governance structures in parallel to specify, pay for and influence these services. The most influential formulation of pro-commodification policies, the Washington consensus (Williamson, 1993), recommends competitive procurement, provider competition and specific forms of pricing as the structures governments should mainly use. Many studies analyse how they operate in the hospital sector (for example Busse et al., 2013; Harrison, 2009; Tan et al., 2014) but few analyse how they affect community health activities provided by voluntary, community and social enterprise (VCSE) organizations. Systematically comparing six localities in England during 2020–2022, we found that two distinct modes of commissioning coexisted. Commodified commissioning largely embodied Washington consensus assumptions and recommendations. Alongside, a contrasting, collaborative mode of commissioning had developed, more attuned to VCSEs’ focus on ‘mission’ and ‘gift’ (Titmuss, 1997). When the two modes conflicted, commissioners often favoured the collaborative mode and sought to adjust their commissioning to make VCSE activity less commodified, partly reversing earlier shifts towards commodifying VCSE activity. This article indicates the characteristics defining commodification, in the light of which it presents some findings from the systematic comparison, and finally some implications, gains and losses of the commissioning of VCSEs.

## Conceptualizing commodification

Many governments have policies of commodifying public services to varying extents. The Washington consensus policy assemblage combines privatizing public providers, marketizing the public sector, private provision of publicly-funded services, and making public sector organization and management literally ‘business-like’. The commodification of interactions between the state and service providers is an integral component.

At the level of whole welfare regimes, commodification has often been understood as: ‘the extent to which workers and their families are reliant upon the market sale of their labour’ (Bambra, 2005; Bergmark, 2008; Christiansen, 2017; Esping-Andersen, 1989). In quasi-markets public services themselves remain uncommodified for users, whether free of charge (as in the National Health Service [NHS]) or with, say, a social insurer reimbursing the user but, behind the scenes, the inputs become commodified through labour market casualization, out-sourcing, corporate finance of new infrastructure, public-private joint enterprises, and the creation of intellectual property. Bambra (2005) therefore proposes an index of healthcare commodification based on the percentages of population whom the public healthcare system serves, of expenditure on private services, and of private providers. More broadly, commodification has been equated with marketization (Tonkens et al., 2013), even capitalist production *per se,* which may be why some writers (Caplan, 1989; Esposito & Perez, 2014; Goldstein & Bowers, 2015) use ‘commodification’ as a condemnatory term and why, in practice, the sale of services may be masked as something else (Lupton, 2014; Norman et al., 2016; Scheper-Hughes, 2001).

Political economists since Marx have conceptualized a commodity as simultaneously being:

- A physical object or service whose physical characteristics, or information or media whose contents, are practically useful to its buyer (Bambra, 2005; Bergmark, 2008; Caplan, 1989; Christiansen, 2017; Esping-Andersen, 1990).
- The property of the person or organization (Carvalho & Rodrigues, 2015) having exclusive *de facto* control of it, at least in the case of private and state (but not communal) ownership.
- Priced, at a level approximating to its production cost including overheads (equipment, transaction costs, rents) plus profit (OECD, 2015). Prices at that level distinguish commodification from (among others) ritual exchange, tribute, taxes, theft, sacrifices, something claimed by right (Mackintosh, 2006), gifts (Titmuss, 1997), charity or self-help (Caplan, 1989).
- Produced for sale, which in practice requires that buyers lack easier ways of obtaining the product or service (for example by making it themselves) and have enough money to buy it at the above price. The Washington consensus and its underpinning micro-economic theory assume that the producer and buyer respectively formulate the product specification and buyer demands independently, although the provider also designs the good to be saleable (Krajewski, 2010; Mazanderani et al., 2013; McClean & Moore, 2013; Stoeckle, 2000). Negotiations between seller and buyer are a zero-sum activity whose outcome depends on the balance of power between the two (Altman, 2015). In a quasi-market the buyer is a third party (such as the clinical commissioning groups described below) rather than the actual service user, and these buyers are structurally separated even from publicly-owned service providers (the ‘purchaser–provider split’).

Commodification can then be defined as bringing goods or services into the specific social relationships listed above; de-commodification is the opposite. Commodification thus has consequences for what services are produced, for whom and to what specifications; who has access to them, which depends on prices, payment-systems and income-distribution; and what is *not* produced. When services become commodified, their character can change.

### Modes of commissioning

**Figure 1.**
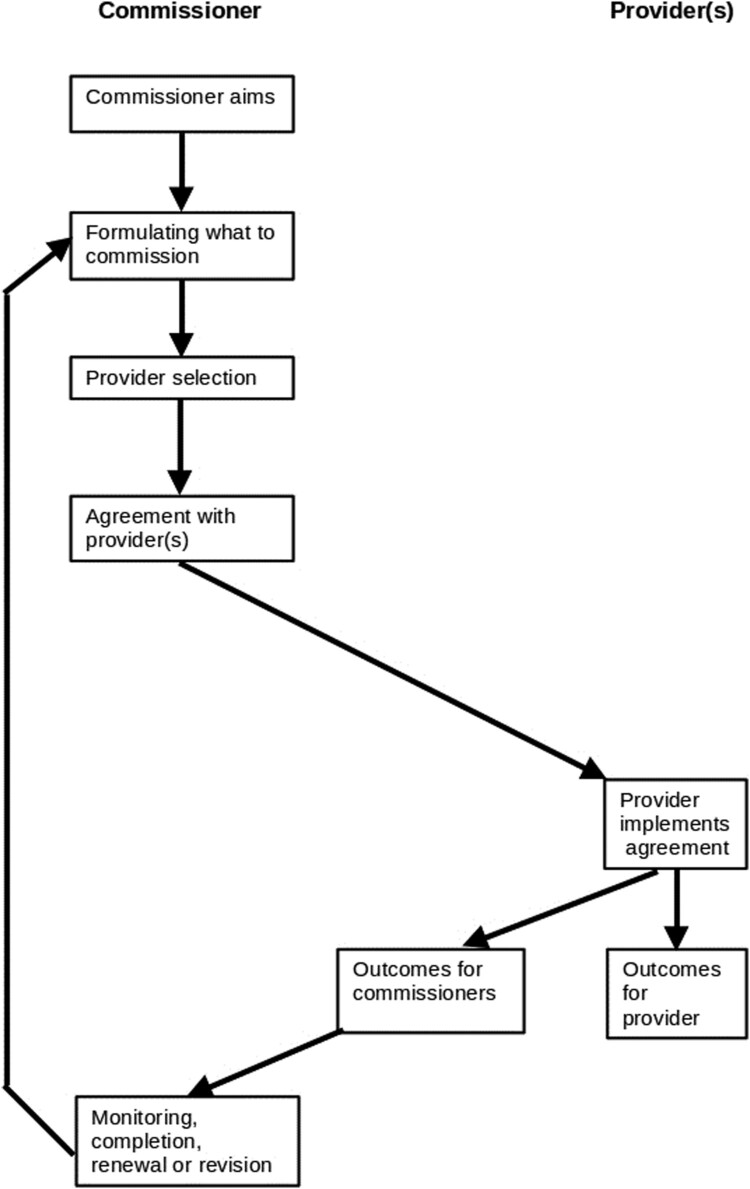
The commissioning cycle.

In quasi-markets the organizations which mediate between the state and service providers commission services through a repeating cycle whose main stages are: formulating what services to commission; provider selection; agreeing a contract with the selected provider(s); contract implementation (which the provider undertakes but the commissioner reviews); contract completion, renewal or variation (Figure 1).

Commissioning organizations use up to six main governance structures in parallel to specify, pay for and influence commodified public services (Sheaff et al., 2015). First, management techniques used for planning and monitoring services within an organization are extended and adapted for managing external organizations quasi-hierarchically (Exworthy et al., 1999; Petsoulas et al., 2011; Vincent-Jones, 2007) through a stable inter-organizational network with the commissioner as its co-ordinating centre. Second, the separate organizations may come to agree (perhaps only implicitly) a negotiated order (Bishop & Waring, 2016): a division of labour through which they co-ordinate, even pool, resources in pursuit of shared activities or aims. Discursive control through normative or scientific persuasion is a third means. Fourth, a commissioner can stipulate the conditions under which it will purchase services (Coupet & McWilliams, 2017) and set up financial incentives. Competition between providers gives commissioners a fifth means of control (Krajewski, 2010; Mazanderani et al., 2013; McClean & Moore, 2013; Stoeckle, 2000). Last, commissioners can legally enforce contractual obligations and ownership rights.

In practice, commissioning organizations select and hybridize media of control according to the local health system’s organizational character, history and geography (Noort et al., 2020), provider ownership (for example public, corporate, third sector) (Chambers et al., 2013), their own policy aims (Albalate et al., 2021), and the type of service. Where services were mostly commodified, one might expect to observe modes of commissioning much as the Washington consensus advocates, with formalized procurement procedures, provider competition, and predominantly contractual relationships between commissioners and providers. Negotiative, managerial, relational and discursive methods of control would be more marginal. Commissioners would rely more upon market and contractual than hierarchical and network governance structures. For largely de-commodified services one would expect to observe commissioners using predominantly negotiative, managerial and discursive methods to control providers, with contractual controls, provider competition and competitive procurement systems being marginal or largely symbolic. Then, commissioners would rely more upon networked governance structures, less on quasi-hierarchical and less again on market and contractual governance structures. One might expect commissioners under tighter fiscal constraints to be more selective about providers, hence more likely to use competition-based control.

### Commodification of community care

Glaser (1987) and Roemer (1993) comprehensively reviewed the then prevalent forms of commodification and commissioning of hospital services. Subsequently the Diagnostic Related Group (DRG) system became predominant (for overviews see Busse et al., 2011, 2013; Leister & Stausberg, 2005; Tan et al., 2014). These forms of commodification and commissioning were the prototypes when commodification was extended to non-hospital healthcare. Studies of commodification in primary healthcare focus mainly on general practice in countries that follow the English or a similar system (Saltman et al. (2006) give an overview). Except in Scandinavia, these forms of commodification still reflect GPs’ historic status as ‘free professionals’ (Bergeron & Castel, 2010) selling their services direct to patients or, nowadays, third-party payers in a quasi-market. However, these forms of commodification appear ill-adapted for co-ordinating services across multiple providers, especially for people with complex, chronic health problems, so there have been numerous experiments with alternatives (among many see Anderson et al., 2015; Briot et al., 2015; Damery et al., 2016; Hildebrandt et al., 2015; and the *International Journal of Integrated Care)*.

Demand pressures on hospitals, not least due to Covid 19, and increasing interest in preventing ill-health have drawn policy-makers’ attention to community care in a wide sense covering both formal services provided in or near the patient’s home (for example home nursing, hospices, social care) and informal care (for example ‘social prescribing’ of preventive self-care, such as outdoor walking or social activities). Policy-makers in the UK and elsewhere are increasingly turning to VCSEs to undertake such activities (Cabinet Office, 2018). In England, both local authorities and the NHS commission VCSEs. A Better Care Fund provides ring-fenced money for the NHS and local authorities to use collaboratively. Regulations mandate service ‘procurement’ by competitive bidding but in some circumstances VCSEs may receive grants. Some service users nominally have a personal budget to spend on whatever community care they choose, although in practice the local authority often administers these budgets. Some, such as those with learning disability, may receive care simultaneously from the NHS, local authority, advocacy support (legally mandated), and VCSEs. The NHS (2019) looked to VCSEs to extend the preventive reach of primary healthcare, provide innovative health maintenance activities, and help make healthcare commissioners more responsive to local demands and needs. These policies have tended to extend commodification further into the relationships between state and VCSEs.

English VCSEs often have difficulty dealing with NHS commissioners’ procurement systems (Allen et al., 2011; Currie et al., 2018) but little is known about how VCSEs’ community healthcare activity becomes commodified when public bodies commission it, how it might change as a result, what modes of commissioning tend to emerge or are adapted to this setting, nor what limits there are to commodification in it. Given the differences in activity (community versus hospital care) and provider ownership (VCSE versus public or corporate), we hypothesize that modes of commissioning in this sector will be at the non-commodified end of the spectrum suggested above. Using new data from England we therefore address the following questions:

- What modes of commissioning develop, and why, for community healthcare activities provided by VCSEs?
- How do these modes of commissioning arise from, and affect, the commodification of these activities?
- What implications follow for the above theories of commodification?

## Methods

Conceiving of commissioning as the main intervention by which commissioners try to influence VCSE community healthcare activities in their localities, and using realist methods, we systematically compared case studies of the commissioning of these activities in six English localities during 2020–2022. We focused on what commissioners wanted to achieve, what mechanisms they attempted to use, and what contexts affected how those mechanisms worked in practice. As the mechanisms, we focused on the media of control mentioned above; on what combinations of them, i.e. modes of commissioning, developed as a result; and how VCSEs responded. Like any other provider, VCSEs were, we assumed, simultaneously trying to become commissioned as a means (mechanism) to achieve *their* own ends.

Our unit of sampling and of analysis was the set of commissioning relationships within the footprint of a Clinical Commissioning Group (CCG), the NHS organizations which, at the start of our study (2020), undertook most healthcare commissioning in England, although local authorities commissioned public health, social care and a number of other health-related activities (for example self-help groups). From 2022, Integrated Care Systems (ICS) replaced CCGs. We sought a purposive maximum variety sample in terms of CCG approaches to commissioning. Assuming that differences in spending patterns might reflect distinct modes of commissioning, we used published financial data to rank CCGs by per capita spend on VCSE activities, and sampled one site (defined by CCG) from each quartile, adding for maximum variety one additional site each from the top and bottom quartiles, making six altogether. These CCGs served populations ranging from about 500,000 to about 1,200,000 (numbers rounded for anonymization). The VCSEs ranged from small local organizations to large national ones. Within sites, we focused on three contrasting tracer activities, also selected as likely to show maximum variety of commissioning practice: social prescribing (community-based, many small, diverse providers); end-of-life care (a few large providers per site); and support for people with learning disabilities (mixed formal and community care, some self-organization, some corporate and public providers).

We collected data (see Table 1) from key commissioner and VSCE informants for each tracer group in each site, and from key informants in NHS and VCSE national bodies. Interviews ranged from 40 to 106  minutes, were audio-recorded and transcribed. We observed commissioning meetings and meetings of the networks described below, and content-analysed administrative documents which our informants said were relevant to the study and consulted previously-published studies.

**Table 1.** Data sources.

National-level informants	13
Site-level: commissioners	61
Site-level: VCSEs	98
Documents	111
On-site project reference group meetings (local advisors)	18

We used a framework analysis to compare sites systematically. It was conceptually equivalent to tabulating the data by the four defining characteristics of commodification (equivalent to virtual rows), and by what media of power hence mode(s) of commissioning were present (equivalent to virtual columns). Following grounded theory principles, we inductively analysed data which did not fit these categories. Finally, we grouped the patterns found by the above research questions. The second research question contained, in part, hypotheses of our own devising, so we looked deliberately for data and patterns of findings that might require us to modify or reject those initial assumptions. Our study had NHS research ethics approval, IRAS reference 270268.

## Findings

We present our findings by the four defining characteristics of commodification. Sites are anonymized as ‘CS1a’, ‘CS1b’ etc, informants as ‘_C1’, ‘_C2’ etc. for commissioners, and ‘_V1’, ‘_V2’ etc. for VCSE informants.

### Specifying the commodity

Commissioners extended their existing procurement systems, originally devised for commissioning hospital care with its corresponding evidence bases, to VCSEs. To varying extents commissioners wanted to specify clearly what activities the commissioned VCSE would provide:

*The CCG or PCTs before that were very much the sort of contractor and commissioner and would be expecting their providers to be delivering against a set of annual objectives and key performance indicators* (CS2b_C8).

One motive was the commissioners’ legal obligation to guarantee certain services or activities. Another was defensive:

*someone could challenge me and could come along and say, ‘I want to see the results of that tender’ … it is a legal process that I must follow … I could be challenged and, so, I’m tied* (CS4a_C2).

The result would be a rigid specification of VCSE activity:

*We won the contract and then they realized they had a political problem. And I said, ‘You’re not ready, we could give some of the contract away, we could do this’ and they said, ‘No, no we’ve been through the procurement process, you’ve got to have it’* (CS2b_V5).

Commissioners’ desires to specify outputs contrasted with the undefined, emergent character of many VCSE activities and outputs. Advocacy was one. Client groups’ demands often could not be known until they arose, for example when commissioners and VCSEs co-designed services:

*We used to have 40 or 50 organizations together in a room and … co-design things like domestic abuse services or whatever it was, we would kind of all get together and look at that together* (CS2b_C11).

Some services, such as information or advice services, could be specified in advance but not who would receive them. Social prescribing and support for people with learning disability often transcended service boundaries:

*We run … employment based projects, mobility projects, all sorts of stuff. And, what’s really important to us is they’re sort of fluid, they overlap, they complement each other* [but] *commissioning dictates that we do things very siloed, very separate to one another* (CS4a_V4).

For other activities the outcomes, although specific, were indirect or gradual. In preventive care they might be that something did *not* occur:

*It is really difficult to evidence that we have stopped people going to their GP by providing a dementia lunch club and it’s really difficult to evidence that we have saved the council or the CCG money by doing our, what we do* (CS3b_V1).

Another hard-to-specify social prescribing outcome was supportive interpersonal relationships between client and volunteer or paid worker:

*take mental health and well-being … sitting down with someone who just comes into a group who wants to come once a week for a cup of tea and a bit of toast or something, are you going to sit down with them every week? We struggle with how we measure what we’re achieving, what the impact is* (CS2b_C11).

Indeed, measurement could undermine the activity itself:

*we said, ‘No! We don’t do pre-engagement questionnaires’ and ask people, you know, what are their underlying health conditions, how often do they visit the GP and stuff. We would never ask that stuff in a million years because that creates a relationship that is immediately deficit-focused, is immediately about us trying to fix people and that’s not what we’re about* (CS4a_V4).

A workaround for these problems was for commissioners to commission VCSE ‘capacity’ to provide services or activities as needed, or resources (for example premises) that supported several services, rather than pre-defined volumes of activities or clients. Had commissioners only commissioned tightly-specified, that is highly-commodified, services the range and scale of funded VCSE activities would have been much smaller.

### Exclusive ownership

Organizational and ownership boundaries did not prevent inter-organizational collaboration among commissioners, among VCSEs, and between commissioners and VCSEs, although they could create barriers.

Commissioners collaborated, for example, in forming ‘a really active carer strategy group etc. which is a broad range of officers from commissioning, adult social care CCG etc. and our partners’ (CS3b_C2), or a partnership board which included ‘people themselves with learning disabilities. Yeah, and police sometimes come, fire have come a couple of times, buses’ (CS1A_C6). Some posts were shared between the CCG and local authority. These horizontal networks in effect created a networked single commissioner for many VCSE activities.

Commissioners’ procurement systems could nevertheless bring VCSEs into competition:

*A lot of the voluntary and community sector in this county tell us that we, commissioners and commissioning, have put them into competition with one another, so they can’t really collaborate to come together to push their own agenda* (CS4a_C2).

The VCSEs tried to attenuate these competitive pressures:

*There are many different social prescribing type services within this space and we’re in competition with some of them, although we try not to be. We try to take an approach that there’s enough work for everybody and we just need to agree on how … we’re going to share that out* (CS3b_V1).

All study sites had networks of collaborating VCSEs co-ordinated through an ‘infrastructure’ or ‘umbrella’ organization, although the strength and number of networks varied between sites. One site had a series of local infrastructure bodies:

*probably three community anchor organizations that are strong, have a turnover of I think over £1 million and are really embedded in their communities … There are, I’d say, probably another four or five who are good community anchor organizations* (CS3b_V1).

Other sites had just one such network.

**Figure 2.**
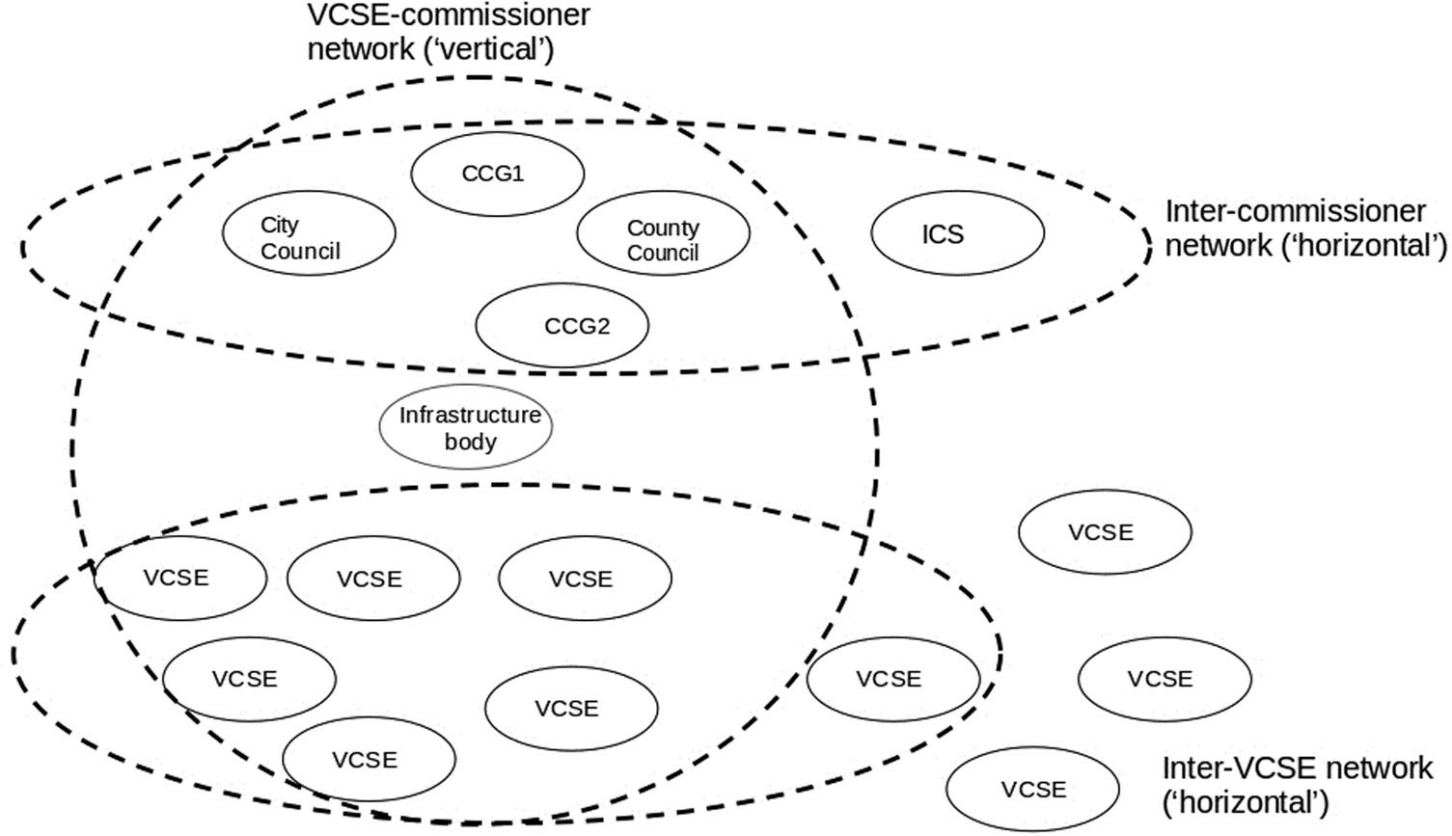
Three commissioning networks.

Alongside the ‘horizontal’ networks of commissioners, and horizontal networks among the VCSEs, ‘vertical’ networks between commissioners and VCSEs also developed, producing the configuration shown in Figure 2.

As noted, commissioners often depended on VCSE input to decide what activities to commission. Vertical networking was achieved partly through formal partnerships, in which local authority officers ‘support voluntary sector organizations to do bids and to support with getting contracts’ (CS4b_C1), and partly by including VCSE representatives in the inter-commissioner networks:

*69 representatives across the voluntary and community sector sitting on various boards across* [site] *that the public sector would set up and ‘own’, quote marks, and then the voluntary and sector would have a representative sit on that board. And my job was to ensure that they were fully representative of the voluntary and community sector, as much as they could be* (CS4a_C2).

The NHS had also begun building VCSE representation into its new ICSs and local (‘place’ level) co-ordinating bodies.

Some VCSEs participated in the vertical networks not as service providers but as advocates for communities of interest, identity or place. These VCSEs argued that their input was valuable precisely because they had no vested interests to defend. Such inputs contributed to the commissioners’ wider commissioning strategies and plans:

*it’s normal if you commission a provider to do something to do with dementia, then that provider then gets a place on that board. So, if you’ve got a dementia work-stream then you’ve probably got Alzheimer’s Society on it because we commission them to do dementia work. Is that the right way around?* (CS4a_C2).

As another commissioner said: ‘I wouldn’t go as far as to say it’s always co-production but it’s an attempt’ (CS1b_C1).

Many of the commissioned activities involved multiple VCSEs, so ‘that’s always been a slight, problem is not the right word, challenge I think is the better way of putting it, into how they could be co-ordinated’ (CS2b_C8). Commissioners themselves would then get drawn into co-ordinating the activities:

*making sure that the scheme is working the way that it should be within the financial envelope … that’s kind of the commissioner’s role but also working with other partners … So, if somebody living in there* [sheltered accommodation for people with learning disabilities] *wants to get a job, then we enable that provider to link in with the local community to get that person a job … it’s not just about contract monitoring, it’s about facilitating that contract as well* (CS1b_C1).

Practical responses to any problems that contract monitoring revealed were also sometimes made in these negotiative, relational ways; sometimes in more formal, transactional ways; but seldom by commissioners replacing one VCSE with another.

Although commissioners often thought that VCSEs would be well-informed contributors to setting service specifications, they also tended to think that the procurement rules prohibited that contribution. In all our sites, VCSEs claimed that bidding for commissions tended to introduce competition and mistrust between VCSEs, which was not only counter-cultural for them but a practical obstacle to VCSE activities which relied on inter-organizational collaboration (for instance supporting people with multiple complex care needs). The vertical networks between commissioners and VCSEs described above:

*brought together commissioning and procurement and I know* [that in] *a lot of places that’s done separately … we had, as you can probably imagine, two very different schools of thought between those two teams, commissioners who are all about kind of working with providers and people and designing services and procurement all about kind of, ‘Well you can’t do that in procurement legislation, you need to avoid the risk of* [legal] *challenge’* (CS1b_C1).

This conflict recurred across sites and care groups.

### Prices

Again, commissioners sometimes ‘specified exactly what we want and we are paying’ (CS3b_C2). Then the pricing unit might be for a specified output (activities), or less clearly specified ‘outcomes’, or ‘capacity’ (see above). One VCSE reported a surplus on its contract but otherwise prices were often below the actual cost of the activity. Overheads were usually partly or wholly excluded: ‘in general, we can’t charge more than 10% to 15% management fee on any of our contracts but our actual central costs are about 25% of our organization’ (CS3b_V1). Some prices did not even cover the direct costs:

*They* [commissioners] *say, ‘We give you £40,000, you provide two hours of activity a week to 140 people’ and … divide the first figure by the second figure … the unspoken thing is that they want the result of that sum to be as close to the national living wage as possible really, like the cost should be £10 per person per hour, and it’s about £20 per person per hour with us* (CS1a_V16).

VCSEs also bore the opportunity cost (activity foregone) of attending commissioning meetings:

*A lot of commissioners don’t realize that by me turning up as a charity rep. at a meeting, I’m not being paid to be there and therefore I’ve got to be saying in my head, ‘Our mission statement is* [details removed for anonymization] *am I doing that here? If not, I’m breaking charity law!’ you know?* (CS2b_V5).

Prices were not necessarily increased to match inflation. In effect, VCSEs sometimes cross-subsidised their commissioned activities from donations, fund-raising, subscriptions, other sales, or investment income.

Some commissioners paid VCSEs by grant when possible ‘because that is another way that you don’t have to do competitive procurement’ (CS2b_C11), especially when, for example, specifying and pricing VCSE activity was difficult or when they wanted to fund VCSEs ‘to pilot things and developing’ (CS2b_C11):

*What we really heard from carers is, ‘I just want to go outside and I can’t go outside’. And then what we did was we put out a call to the voluntary and community sector and said, ‘Based on this, how do you think you could work with carers to get them out into nature?’ And then we had responses and then we’ve given grant agreements to organizations to say, yes. And what they’re doing is they’re co-producing their delivery with carers* (CS4a_C2).

Grants were not necessarily larger or more stable than contracts: the commissioners in one site replaced a five-year contract with one VCSE with an annual grant. Neither did grants necessarily cover the full cost of activities:

*We’ve argued the contribution to young carers service is only a contribution, it’s approximately a third of what they pull in from other places* (CS3b_C2).

Another workaround was:

*Letting them* [VCSEs] *reduce the numbers of people that they’ve got in their grant … so when somebody leaves* [care] *they can bring somebody else in on a spot* [contract] *and we’ll get them that money back and a bit more* (CS1a_C6).

Whether contract or grant, income instability worried many VCSEs, although a minority of them still preferred a contract because that stated clearly what was expected of them and of the commissioner.

Paradoxically, one of the extreme forms of commodification, that of giving clients or carers a personal budget, offered another way of working around the procurement system because then the personal budget holder could choose their service provider without competitive tendering.

### Production for sale

Neither commissioners nor VCSEs were profit-motivated. Commissioners aimed to implement national (and for local government, local) policy including fiscal restrictions, but also manage service overload, prevent political embarrassments and, during 2020–2022, respond to Covid 19. The VCSEs pursued the ‘missions’ of filling gaps in local public services, advocacy, or both. Commissioning them, however, was accomplished through procurement systems based on those designed for highly commodified relationships between commissioner and provider, in which transactions were extensively documented to pre-empt legal action by unsuccessful bidders and prevent providers skimping on their side of the contract.

Applied to VCSEs, commodified procurement systems sometimes had perverse consequences. Providers had to be selected for the quality of bid, rather than the activities they seemed likely to provide:

*The people that would be really good at delivering this service … didn’t put in a very good bid. Somebody else put in a better bid. It was a failing provider, they were in serious measures with them in another bit of their business, and legally* [we] *had to award them more business over somebody that we knew was well set up to do it and could do it* (CS1a_C6).

Commodified procurement systems also imposed problematic transaction costs, which were:

*tough and very resource intensive often for things that are* [say] *£70,000. So just we haven’t got the time to be taking that out to the market* (CS1_C6).

This was especially so when services were fragmented:

[Name] *authority youth service … we had 12 different specifications for each district which were jointly approved by 12 locality panels made up of the district and council members so we did two meetings with each of those panels, so we did 24 meetings over the period of two months … getting each set of local members to agree the specification* (CS2b_V5).

VCSEs often found procurement systems unintelligible. One commissioner had numerous meetings with their VCSEs to explain where the procurement system was flexible and where (and why) it was not, but that was exceptional. Another attempt to mitigate these problems was by introducing standardized framework contracts to reduce transaction costs:

*We’ve got a framework with about 35 providers on and we will go to them and say, ‘Right, we’re opening this new community cluster scheme, it’s going to be six bungalows, this is what the model is, bid for it if you’re interested’ and then you’ll look at the bids and appoint the best one* (CS1b_C1).

More fundamentally, some VCSE informants feared that commodified procurement might favour corporate bidders:

*They* [commissioners] *have to go in and run a procurement process which could quite easily enable Serco* [a for-profit corporation] *to say, ‘We can do that for 7.3% cheaper’* (CS2b_V5).

Some informants also feared that commodified procurement would marginalize small local VCSEs. When commissioners wanted to make VCSE activities uniformly available:

*They come to the conclusion that then it can only be a county-wide service, rather than a local service* [so] *the only contracts that go out are massive contracts and they go to multi-million pound VCS organizations that have no connection to community whatsoever, they might as well be a public sector arm* (CS4a_V4a).

To work around that problem one commissioner tailored the service specifications, stipulating that providers:

*must evidence your local footprint, your ties to the community, your understanding of the young people in the area, which immediately disadvantaged your out-of-county provider coming in and going, ‘We can do that there’, unless they were working with a local provider* (CS2b_V5).

Some VCSEs also asserted that commodified procurement changed the character of their activities, because:

*As soon as people get excited, they want to come together, they want to do something, they get hit by bureaucracy, they get hit by formalization. When they need to apply for a little bit of funding to get something going, it could be for something as little as a laptop, they find out they’ve got to be constituted, they’ve got to have policies, they’ve got to have governance in place* (CS4a_V4a).

Increasing the scale of commissioned activity could also compromise its voluntary character:

*Community activity is usually, traditionally, participated in* [by] *people who not only take from it but have value to bring to it … But, people kept coming in and in and in and they came on the basis that they were there to consume the activity. They didn’t volunteer. … Nobody else baked a cake, nobody else brought anything in, nobody else helped with the bingo or putting the tables out and eventually the nurses said, ‘We can’t do it any more’ and that closed* (CS4a_V4a).

More radically, ways were found to move VCSE activity outside the procurement system as far as possible. Umbrella organizations afforded the opportunity to use ‘lead provider’ or ‘alliance’ contracts. Commissioners would contract just one VCSE (for example the ‘umbrella organization’) which would then distribute the money and activity to others in a more grant-like way. More recently commissioners and VCSEs awaited the changes in procurement rules announced in recent (2021) national policy documents which they expected to permit direct awards to VCSEs without competition.

## Discussion: Commodified and/or collaborative commissioning

Our findings suggest that two contrasting modes of commissioning VCSEs coexist. *Commodified* commissioning, which our informants called ‘procurement’, was mandated by law, regulations and national policy. It maintained commissioner-VCSE separation as bodies with potentially conflicting interests and treated provider competition, in this case among VCSEs, as normal, even desirable. Informal relationships between commissioners and VCSEs were seen as supplementing or facilitating essentially contractual relationships. The contrasting mode was *collaborative* commissioning. It often concerned broadly defined activities whose exact specification would often only emerge *ex post*. It rested upon inter-organizational networks and relationships which crossed and overlapped commissioners and VCSEs, and which contributed especially to contract formation, service provision and development. VCSEs tended to collaborate rather than compete and were motivated by charitable aims not sales or profit-seeking. They often provided activities at below market cost. Contracts documented and supplemented essentially negotiative, collaborative relationships between the VCSEs and commissioners. The two modes coexisted but collaborative commissioning predominated when commissioners were reviewing population needs, service development, and the services currently provided; commodified commissioning while contracts were being drafted and providers were being selected. Collaborative commissioned predominated in respect of integrated, systemic services and activities, but commissioning discrete, episodic, urgent and statutorily-mandated services sometimes involved commodified elements (for example legally binding agreements to guarantee urgent support for vulnerable people). Table 2 summarises how the two modes of commissioning VCSEs contrasted across the four defining characteristics of the commodity.

**Table 2.** Commodity characteristics across two modes of commissioning.

	Commodified commissioning	Collaborative commissioning
Physical characteristics	Defined by client, act or outcome, as far as possible	Extended to advocacy, relational and infrastructural activity
Property rights	Exclusionary, competitive, contractual	Permeable, waived or absent
Pricing	Partly or wholly per client or per activity	Options of block contracts, grants, gifts, subsidies
VCSE motivation	Income, certainty	Income, influence, advocacy

In either case, a ‘real’ activity was commissioned, but the fluid, emergent character of much VCSE activity (to which collaborative commissioning was adapted) conflicted with the more complete specifications that commodified procurement required. Mutual separation between commissioners and providers, and provider competition, tended to conflict with commissioners’ reliance on VCSE contributions to information-sharing, system planning and development, and VCSE advocacy of under-served care groups’ interests. Its fluid, emergent character also often made it hard to establish precise, granular prices for VCSE activity. To use prices as financial incentives, the theoretically ideal pricing unit would be health status, care outcome or prevention of ill-health (Vlaanderen et al., 2019) but in community care that is technically very difficult to define. In any case VCSE ‘prices’ seldom approximated to the actual costs of their activity. Hard-to-specify community care was often more simply and flexibly financed through grants than per-unit prices. As for production for sale, periodic re-tendering threatened the financial sustainability of some VCSEs. Advocates of commodified commissioning might, however, see that positively, as selection for efficiency and innovation. Any form of commissioning requires information collection and documentation, and to that extent transaction costs, but for many of the VCSEs we studied, especially small ones, the transaction costs of becoming commissioned were prohibitive. For all four characteristics that define commodification, commissioners tended to make workarounds that resolved the conflicts mostly in favour of collaborative, not commodified, commissioning.

In summary, the gains from commodified commissioning of VCSEs included clear specification of commissioners’ and VCSEs’ responsibilities. Activity prices below the full cost of provision were a gain to commissioners but a loss to VCSEs. The losses were bureaucratization and transaction costs (especially for small VCSEs), and, when VCSEs had to compete, dis-integration of cross-VCSE activities.

### Just one instance?

These findings are inferred from one country and sector. Data were mostly collected before the NHS had finished establishing its new ICSs. Currently (2023), the UK government is proposing to relax the legal requirements for NHS commissioners to use competitive procurement (Osipovič & Allen, 2021). It remains to be seen what difference these changes will make. Some of the above patterns are, however, reported elsewhere. Collaborative commissioning, with formal medical representation within the commissioning bodies, occurs in primary care in England, Germany and elsewhere. In Germany, the Netherlands and the USA, payment ‘bundling’, ‘year of care’ and ‘disease management’ payments have been piloted as a ways to reverse the dis-integrating effects of fragmented, commodified payments in primary and community care (Bakker et al., 2012; Kifmann, 2017). Commissioners play a role in inter-provider co-ordination in the Netherlands and Sweden besides England (Noort et al., 2020). Christiansen (2017) also describes increased transaction costs of commodified services. Commodification in the commissioning of VCSEs has occurred in many countries, so to that extent the findings may be qualitatively generalizable.

### Implications for theories of commodification

The multiple, recurrent workarounds for commodified commissioning would appear another instance where workarounds symptomatize built-in defects in a managerial system (Bar-Lev, 2015; De Bono et al., 2013; Lalley & Malloch, 2010; Vogelsmeier et al., 2008). Rather than designing and marketing activities for the purpose of profit-making in quasi-markets, as in the cases that Krajewski (2010), Mazanderani et al. (2013), McClean and Moore (2013) and Stoeckle (2000) describe, many VCSEs in our study used quasi-markets as one way, often not the main way, of resourcing activities that they already undertook for other reasons. VCSEs’ pursuit of commissioned work was not usually instance of commodity production (or profit-seeking) masked as something else (Lupton, 2014; Norman et al., 2016; Scheper-Hughes, 2001) but the reverse. To that extent, standard microeconomic explanations and justifications of full-cost pricing appear inapplicable to VCSEs. In our study sites both commissioners and VCSEs saw the purpose of advocacy as representing users’ interests, which calls into empirical question the assumption that commodifying services strengthens providers’ consumer orientation (OECD, 2015), at least in the case of VCSEs.

Our initial account of modes of commissioning also required refinement. Despite conflicting in many ways commodified commissioning and collaborative commissioning coexisted. Within collaborative commissioning, not all media of control were equal. The obscurity of procurement language and systems to many VCSEs suggests that a common discourse—in the sense of consensus between VCSEs and commissioners about what VCSEs are, what their activities should be, and how they ought to be commissioned—is a foundation for persuasive and negotiative control. In the English health system, this foundation is still incomplete. Inter-organizational networks linking commissioners and VCSEs were also important means of establishing a negotiated order. In a process of mutual adjustment, commissioners and VCSEs influenced each other reciprocally. The activities reported above adapted, attenuated and worked around commodified commissioning so that VCSEs could more readily pursue, and commissioners benefit from, VCSEs’ non-commercial ‘missions’.

After the 1990s much VCSE activity in the English health system shifted from a gift to a commodity basis. As VCSEs’ role in the English health system had increased, however, the prevalence and character of the workarounds which commissioners and VCSEs have devised for managing the tensions between commodified and collaborative commissioning suggest that at least a partial shift back again is occurring. Collaborative workarounds of commodified commissioning also occur in (at least) the German and Italian health systems. Insofar as it occurs more widely, the main implication of above pattern of gains and losses from commodified commissioning is that they stem not just from local institutional factors but from the nature of commodification itself.
